# Allport's Registering Dental Ledger

**Published:** 1858-04

**Authors:** 


*Allport's Registering Dental Ledger.*—Within the last five or six years, several admirably arranged Case books for dentists have been gotten up; and quite recently, C. Scott & Co., of Chicago, Illinois, have published one after the plan of Dr. Allport, combining the advantages both of a day-book and ledger. The left half of each page contains two diagrams of the teeth, with a set of hieroglyphic marks indicating nearly all of the operations performed by the dentist upon these organs; while the right half is arranged for entering the charges made for each operation, something in the form of ledger. A dentist, by registering his operations in a book of this kind, is enabled, at any future period, to designate those which he has performed from the operations of other dentists, who may have operated upon the teeth of the same patient.

The Register of Dr. Allport is not only very simple, but also very complete in its arrangement, combining, indeed, all the advantages which any one could desire in a book of this kind.

